# Effects of (−)Epicatechin on the Pathology of APP/PS1 Transgenic Mice

**DOI:** 10.3389/fneur.2014.00069

**Published:** 2014-05-09

**Authors:** Yue-Qin Zeng, Yan-Jiang Wang, Xin-Fu Zhou

**Affiliations:** ^1^Key Laboratory of Stem Cells and Regenerative Medicine, Institute of Molecular and Clinical Medicine, Kunming Medical University, Kunming, China; ^2^Department of Neurology, Centre for Clinical Neuroscience, Daping Hospital, Third Military Medical University, Chongqing, China; ^3^School of Pharmacy and Medical Sciences, Sansom Institute, University of South Australia, Adelaide, SA, Australia

**Keywords:** Alzheimer’s disease, Aβ, inflammation, (−)epicatechin, APP/PS1 transgenic mice

## Abstract

**Background:** Alzheimer’s disease (AD) is a multifactorial disorder characterized by the progressive deterioration of neuronal networks. The clearance of Aβ from the brain and anti-inflammation are potential important strategies to prevent and treat disease. In a previous study, we demonstrated the grape seed extract (GSE) could reduce brain Aβ burden and microglia activation, but which polyphenol plays a major role in these events is not known. Here, we tested pharmacological effects of (−)epicatechin, one principle polyphenol compound in GSE, on transgenic AD mice.

**Methods:** APP/PS1 transgenic mice were fed with (−)epicatechin diet (40 mg/kg/day) and curcumin diet (47 mg/kg/day) at 3 months of age for 9 months, the function of liver, Aβ levels in the brain and serum, AD-type neuropathology, plasma levels of inflammatory cytokines were measured.

**Results:** Toward the end of the experiment, we found long-term feeding of (−)epicatechin diet was well tolerated without fatality, changes in food consumption, body weight, or liver function. (−)Epicatechin significantly reduced total Aβ in brain and serum by 39 and 40%, respectively, compared with control diet. Microgliosis and astrocytosis in the brain of Alzheimer’s mice were also reduced by 38 and 35%, respectively. The (−)epicatechin diet did not alter learning and memory behaviors in AD mice.

**Conclusion:** This study has provided evidence on the beneficial role of (−)epicatechin in ameliorating amyloid-induced AD-like pathology in AD mice, but the impact of (−)epicatechin on tau pathology is not clear, also the mechanism needs further research.

## Introduction

Alzheimer’s disease (AD) is a neurodegenerative disorder that primarily strikes the elderly. Clinically, AD patients develop symptoms consisting of a gradual loss of memory, mental confusion, language disturbances, personality and behavioral changes, and diminished abilities for reasoning, orientation, and judgment. According to the widely accepted “amyloid hypothesis” (1), the overproduction of Aβ in the brain, or failure of Aβ clearance lead to brain deposition of Aβ and a series of secondary pathological events, such as neurofibrillary tangle formation, neuronal dysfunction, and microglia activation, which characterize affected brain of AD (2, 3).

Aβ is regarded to play pivotal or causal roles in the development of AD, therapeutic strategies have been mainly focused on reducing Aβ production, inhibiting Aβ deposition, and promoting Aβ clearance (4). Accumulation of Aβ in the brain and development of AD have been linked to dietary factors. Polyphenols are a type of antioxidants presenting in plant-based foods and confer significant health benefits, including protection against AD and other memory problems (5, 6).

Our previous studies have shown that polyphenols in the grape seed extract (GSE) could prevent the Aβ deposition and attenuates the inflammation in the brain of a transgenic mouse mode (7). GSE, which contains various polyphenols including gallic acid, catechin, EGCG, EGC, epicatechin-3-gallate, epicatechin, and proanthocyanidins (8), but which polyphenol plays a role in AD pathology is not clear. As (−)epicatechin is one of a major component of polyphenols from GSE (7, 9), we investigate whether (−)epicatechin is effective in AD treatment. Here, we report the beneficial effects of (−)epicatechin diet consumption on the neurodegenerative process of AD mice. Meanwhile, curcumin was used as a positive control, which can clean Aβdeposition and ameliorate inflammation in brain of AD mice (7).

## Materials and Methods

### Animals

APP/PS1 transgenic mice were provided by Jackson Laboratories. These mice were constructed on a C57BL/6 background and bear a chimeric mouse/human (Mo/Hu) APP695 with mutations linked to familial AD (KM 593/594 NL) and human PS1 carrying the exon-9-deleted variant associated with familial AD (PS1dE9) in one locus under control of a brain- and neuron-specific murine Thy-1 promoter element (10). They were bred in standard animal house (at approximately 8 weeks of age, male C57BL/6 mice and female APP/PS1 mice breeding pairs, or female C57BL/6 mice and male APP/PS1 mice were housed together as breeding pairs). Genotypes were determined by PCR following the provider’s instruction. Mice were housed individually in standard cages with a reversed 12-h day/night cycle and provided commercial pellet diet and water *ad libitum*. And then, we got enough number of animals for the research.

At the age of 3 months, APP/PS1 mice were divided into three diet groups, (−)epicatechin group (*N* = 12, half males and half females, fed with (−)epicatechin diet), curcumin group (*N* = 12, half males and half females, fed with curcumin diet) and normal diet group (*N* = 12, half males and half females, fed with normal diet). Another age and sex matched wild-type littermates (*N* = 12, half males and half females, fed with normal diet) were used as a control group.

All the animals were treated in strict compliance with the United States Public Health Service Policy on Human Care and Use of Laboratory Animals, the National Institutes of Health Guide for the Care and Use of Laboratory Animals, and the Guidelines for the Care and Use Laboratory Animals of the Kunming Medical University. The study had been approved by the Committee for Animal Experiments and Ethics at the Kunming Medical University.

### Dietary intervention

All experimental diets were prepared by Zoopery Centre, Kunming Medical University. The control diet was the standard commercial pelleted diet (Beijing KeaoXieli Feed Company, Beijing, China). The groups of transgenic mice treated with (−)epicatechin and curcumin (>95% purity by HPLC, Shanghai Tauto Biotech Co., Ltd.), respectively received doses of 40 and 47 mg/kg/day based on previous reports (11, 12). The animals were fed with the above diets for 9 months starting 3 months age, when no Aβ plaque deposition is detectable in the brain. Food consumption and body weight data were collected every 3 months throughout the study.

### Morris water-maze test

After 9 months of dietary treatment, a Morris water-maze test was used to analyze memory capability of these mice. In brief, the apparatus consisted of a circular water tank (100 cm in diameter and 40 cm in height, filled with water in a depth of 30 cm, at 21 ± 1°C) with a platform (11 cm in diameter) set under the water (1 cm below the water surface). The mice must learn to escape from water and step onto the platform. In this study, the mice were trained seven consecutive days with three trials per day. For each trial, each animal was put into the water at one of three starting positions of non-platform quadrants, respectively, and allowed at most 60 s free swim. The escape latency (test duration), path length (total distance traveled), and swim speed (average speed) will be recorded. (If the animal finds the platform within 60 s, it should be stayed for an additional 30 s on the platform; or, it should be gently guided on the platform, and allowed to stay for 30 s). On day 8, a probe trial was performed to test retention of the task. The platform was removed and each mouse was allowed 60 s free swim at a starting point far from the platform (starting point B). The time animal swim in the target zone, the number of crossings over the platform, and the swim track were recorded semi-automatically by the Anymaze video tracking system (Stoelting Co., USA).

### Tissue sampling and preparation

At the end of the experiment, the mice were deeply anesthetized with the lethal concentration of inhaled ether, the blood was sampled from the right atrium of the heart, and then perfused with 50 ml of normal saline, the brains were removed and bisected in the mid-sagittal plane, Left brain hemisphere was fixed in 4% paraformaldehyde (pH 7.4) for 24 h and incubated for 24 h in 30% sucrose, coronal sections of the brain were cut at 35 μm thickness with a cryosectioning microtome and stored at 4°C in PBS containing 40% glycol for histological quantitative analysis and the right brain hemisphere was snap frozen in liquid nitrogen and stored at −80°C until biochemical analysis.

### AD-type pathology and quantitative image analysis

The left brain hemisphere was processed according to the free-floating immunohistochemistry protocol as described previously (13). Briefly, a series of five equally spaced tissue sections, spanning the entire brain were selected and stained using free-floating immunohistochemistry for total Aβ (Biotin-conjugated mouse anti-Aβ antibody 6E10, Serotec; 1:1000 dilution), activated microglia (rat monoclonal anti-CD45, Millipore Bioscience Research Reagents; 1:1000 dilution), and astrocyte (rabbit polyclonal anti-glial fibrilliary acidic protein, Dako, Denmark; 1:1000 dilution), respectively. Sections were incubated overnight with primary antibodies at 4°C and the reaction products were visualized with diaminobenzidine (No. AB500-500 Slide Kit Chemical International, Inc., Millipore).

The region of neocortex and hippocampus manually was selected for quantification of total Aβ plaques, microgliosis, astrogliosis, and microhemorrhage. All images were acquired in the same session. Images were collected at 4× magnification using constant bulb temperature and exposure, yielding the area fraction of the total positive staining against the area of tissue analyzed.

Microhemorrhage (MH) staining and quantification were performed with the described method: in brief, the sections were stained with Prussian blue working solution (equal parts of freshly made 5% potassium ferrocyanide and 5% hydrochloric acid) for 30 min at room temperature, washed in deionized water, and counterstained with nuclear fast red. MH events in the form of the number of Prussian blue-positive profiles were counted in the brains of each mouse on all sections under microscope, and the average number of hemosiderin deposits was calculated per each brain hemisphere. All image analyses were processed in a blind manner.

### Biochemical assay

#### Quantification of Aβ peptide levels in the mouse brain and plasma

ELISA analysis of the brain Aβ was processed as described previously (14). Briefly, frozen brain was homogenized and sonicated in TBS containing protease inhibitors. Homogenates were centrifuged at 100,000 *g* for 1 h at 4°C, and the resultant supernatant was collected, representing the TBS-soluble fraction (Aβ-TBS). The resultant pellet was suspended and sonicated in water containing 2% SDS and protease inhibitors. The SDS solubilized homogenates were centrifuged at 100,000 × *g* for 1 h at 4°C, and the resultant supernatant was collected, representing the SDS-soluble fraction (Aβ-SDS). The resultant pellet was then extracted in 70% formic acid (FA) and centrifuged, and the resultant supernatant was collected, representing the SDS-insoluble fraction (Aβ-FA). Before ELISA assay, formic acid extracts were neutralized by 1:20 dilution into 1 M Tris phosphate buffer, pH11, and then diluted in sample buffer. Concentrations of Aβ40 and Aβ42 in brain extract and serum were quantitatively measured by ELISA according to the manufacturer’s instructions (Cat. #EZBRAIN 40 and Cat. #EZBRAIN 42, Millipore). Using the wet weight of brain tissue in the original homogenate, the final values of brain Aβ were expressed as picograms per gram wet weight of brain.

#### Quantification of TNF-a, IL-1β, and IFN-γ in the mouse plasma

Tumor necrosis factor alpha (TNF-α), interleukin-1β (IL-1β), interleukin-6 (IL-6), and interferon-γ (IFN-γ) in the plasma of mice were measured using ELISA kits (Cat No.BMS607/2, BMS6002, BMS606, BMS603/2 eBioscience, USA) as per manufacturer’s instructions.

#### Assessment of toxicity of (−)epicatechin

Total bilirubin, alanine aminotransferase (ALT), and aspartate aminotransferase (AST) were analyzed by Clinical Laboratory of First Affiliated Hospital of Kunming Medical University.

### Statistical analysis

Unless otherwise stated, the data in the text and figures are expressed as mean ± SEM. Statistical comparisons between groups were analyzed using *t*-test, one-way ANOVA, or two-way repeated-measures ANOVA for testing the significance of values. If significant, *post hoc* testing was done with Tukey’s HSD or Dunnett’s T3 methods, and appropriate *P* values are reported based on adjustment according to Levene’s test for equality of the variance. All these analyses were performed using SPSS 17.0.

## Results

### (−)Epicatechin is well tolerated in APP/PS1 transgenic mice

**Figure 1:**
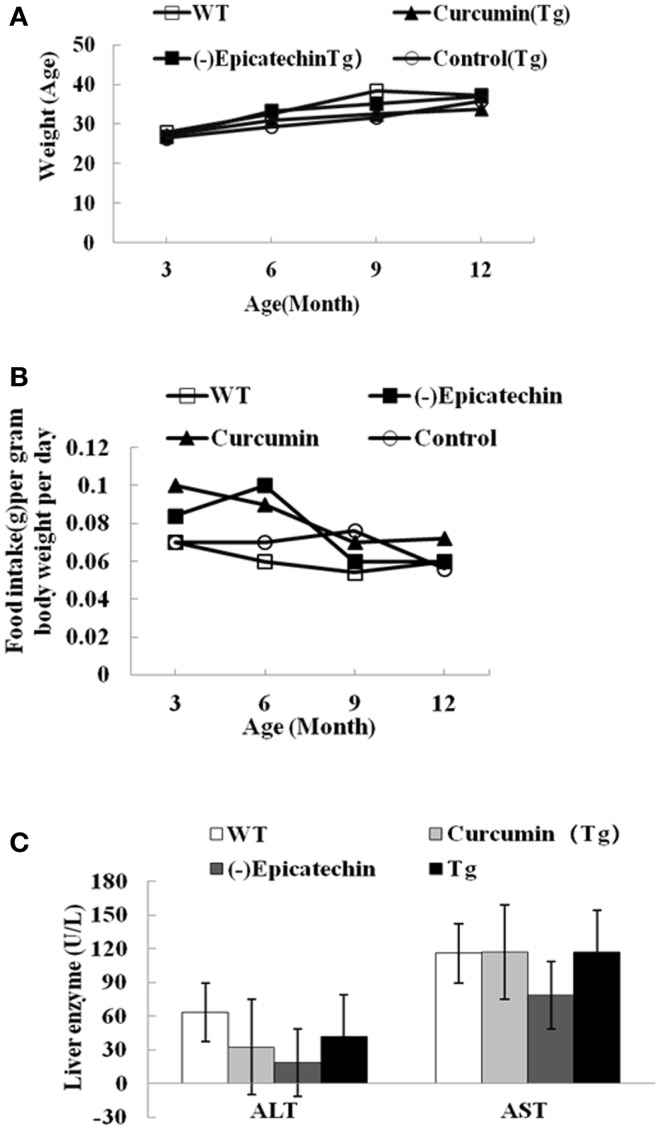
**(−)Epicatechin is well tolerated in APP/PS1 transgenic mice**. APP/PS1 transgenic mice were fed control, curcumin or (−)epicatechin diets for 9 months from 3 months of age. In parallel, wild-type littermates were fed with control diet too. **(A)** Body weight was measured at 3, 6, 9, and 12 months of age. **(B)** Food intake was monitored at 3, 6, 9, and 12 months of age, and was calculated as food intake (gram) per gram body weight per day. **(C)** Serum indices of liver functional status such as AST and ALT. Points and bar graphs represent group mean (±SEM).

We started to feed (−)epicatechin and curcumin diet at 3 months of age. The mean daily food consumption of the mice was 0.10–0.12 g/g body weight, the corresponding daily (−)epicatechin consumption was 40 μg/g body weight and daily curcumin consumption was 77–98 μg/g body weight. The equivalent consumption in a 60 kg human is about 0.14 g/day for (−)epicatechin and 0.35 g/day for curcumin, as derived using FDA criteria for converting drug equivalent dosages across species, based on body surface area [human equivalent dose in milligrams per kilogram = animal dose in milligrams per kilogram × (animal weight in kilograms per human weight in kilogram)^0.33^](15). No difference between APP/PS1 transgenic mice and wild-type animals was found in animal viability, body weight, general activities (Figure 1A), daily food consumption (Figure 1B), and liver function, which can be reflected by the normal serum levels of bilirubin, AST, and alanine aminotransferase aspartate (ALT) (Figure 1C, the data of bilirubin was not shown because the level was below detection).

### Effects of (−)epicatechin on learning and memory in APP/PS1 transgenic mice

**Figure 2:**
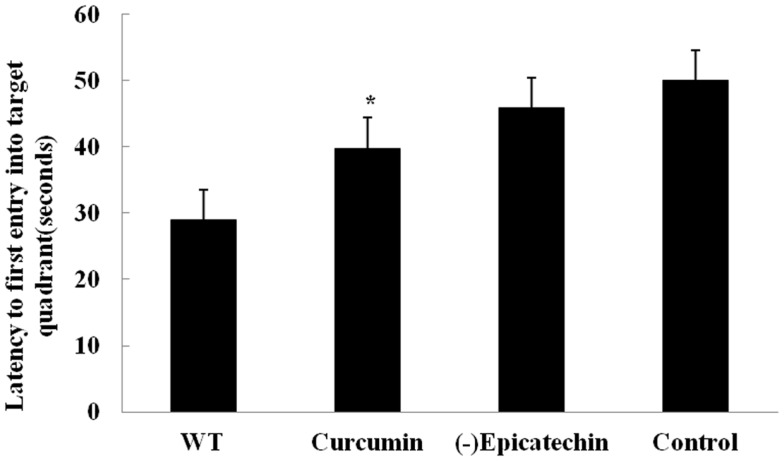
**Effect of (−)epicatechin on learning and memory in APP/PS1 transgenic mice after treated 9 months, Mice were trained for 7 day in Morriz water maze**. On the 8 day performed a probe test, and the time to enter the correct quadrant where the platform had been was measured. One-way ANOVA with Student–Newman–Keuls *post hoc* corrections in this graph, no differences (*P* > 0.05). Bars represent mean ± SEM. Statistical software package was SPSS17.0.

After 9 months treatment, all animals received behavior test. Mice were trained for 7 day with three trials per day to find the hidden platform in the Morris water maze. On day 8, mice were undergone a probe trial in which the platform was removed and the time to enter the correct quadrant where the platform had been was measured (Figure 2). But acquisition of the task did not differ between the groups (ANOVA *F* = 2.004, *P* > 0.05), (−)epicatechin mice did not perform well on the probe trials, indicating that the (−)epicatechin did not enhance retention of spatial memory.

### (−)Epicatechin reduces Aβ levels in the brain and serum and reduces amyloid deposition in APP/PS1 transgenic mice

To assess the effect of (−)epicatechin on amyloid protein levels in the brain and the serum, the levels of both Aβ40 and 42 in brain homogenates and serum were analyzed by specific sandwich ELISA. Aβ in the SDS fraction represents soluble fraction and the diffuse Aβ plaques, and Aβ in the formic acid fraction represents the fibrillar Aβ plaques. Total Aβ level was generated from the sum of Aβ40 and 42 of the different fraction.

**Figure 3:**
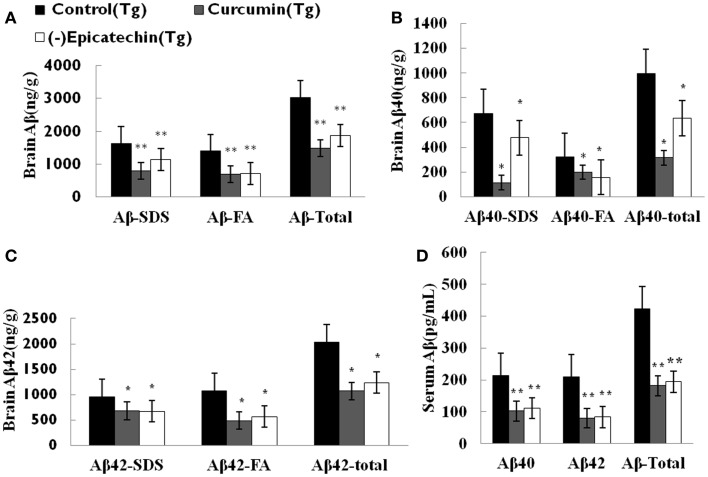
**Effects of (−)epicatechin consumption on Aβ levels in the brain and serum of APP/PS1 transgenic mice**. Aβ peptide concentration in the brain and serum of each animal was measured using ELISA. **(A)** Comparison of total Aβ, Aβ in SDS fraction (Aβ-SDS) and Aβ in formic acid fraction (Aβ-FA) among groups. **(B)** Comparison of total Aβ40, Aβ40-SDS and Aβ40-FA. **(C)** Comparison of total Aβ42, Aβ42-SDS and Aβ42-FA. **(D)** Comparison of total Aβ, Aβ40 and Aβ42 in serum. * and ** denote *P* < 0.05 versus APP/PS1 transgenic mice fed with control diet.

We found significant reduction in total Aβ burden in the brain of mice consuming (−)epicatechin (*P* < 0.05) and curcumin diet (*P* < 0.01) when comparing to Control group (ANOVA *F* = 8.823, *P* < 0.01), (−)epicatechin and curcumin diet reduced total Aβ burden by 39 and 51% respectively (Figure 3A). Similarly, Aβ levels in 2% SDS extracts of (−)epicatechin and curcumin diet groups were also reduced by 37 and 68%, respectively, and the levels in the insoluble pellet in formic acid extracts were reduced by 39 and 47%, respectively (Figures 3B,C, ***P < 0.01). The concentration of total Aβ in the serum was also assayed by ELISA kit, the Aβ level in the serum tended to be lower than that of the normal diet control group (Figure 3D), and the data did not show significant correlation with brain total Aβ burden (Pearson *r* = 0.644, *P* = 0.554).

**Figure 4:**
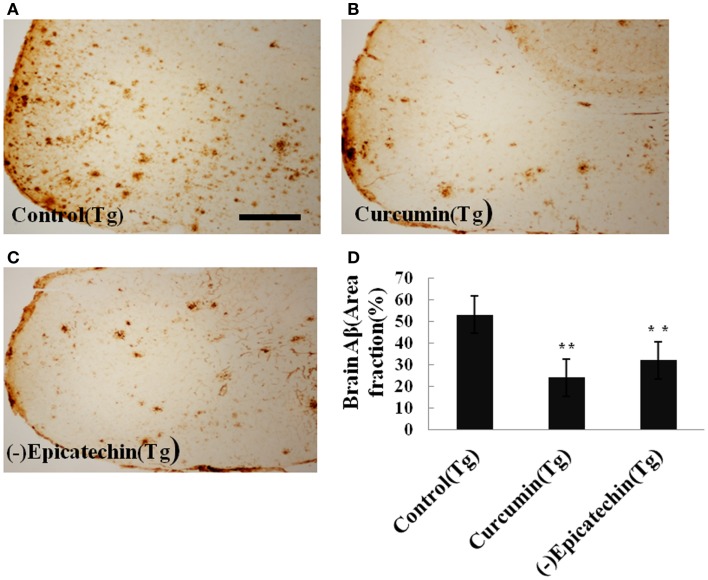
**Effects of (−)epicatechin diet on Aβ deposits in the brain of the APP/PS1 transgenic mice**. **(A)** Aβ plaques of in neocortex of APP/PS1 transgenic mice fed with control diets; **(B)** fed with (−)epicatechin; **(C)** fed with curcumin; **(D)** comparison of Aβ plaque area fraction in neocortex and hippocampus among groups. Statistical analysis showed a significant decrease in the number of plaques in the brain treated with (−)epicatechin and curcumin diets. *P* < 0.05 versus APP/PS1 transgenic mice fed with control diet. Scale bar = 0.5 mm.

Amyloid plaques on coronal sections (35 μM thick) of the neocortical and hippocampal areas were detected with immunohistochemical staining (by CD6E10 antibody). The numbers of Aβ deposits were counted. Quantitative analysis showed a significant reduction of Aβ deposits in mice consuming (−)epicatechin (*P* < 0.05) and curcumin diet (*P* < 0.01) compared with control diet consumption (ANOVA *F* = 5.310, *P* = 0.019) (Figure 4). The result is in agreement with the brain Aβ levels of ELISA analysis. The total Aβ plaques detected by immunohistochemistry was reduced by 40% in the (−)epicatechin diet group and 55% in the curcumin diet group. These results imply beneficial effects of (−)epicatechin and curcumin, which could reduce amyloid protein aggregation.

### (−)Epicatechin prevents AD-type neuropathology in APP/PS1 transgenic mice

**Figure 5:**
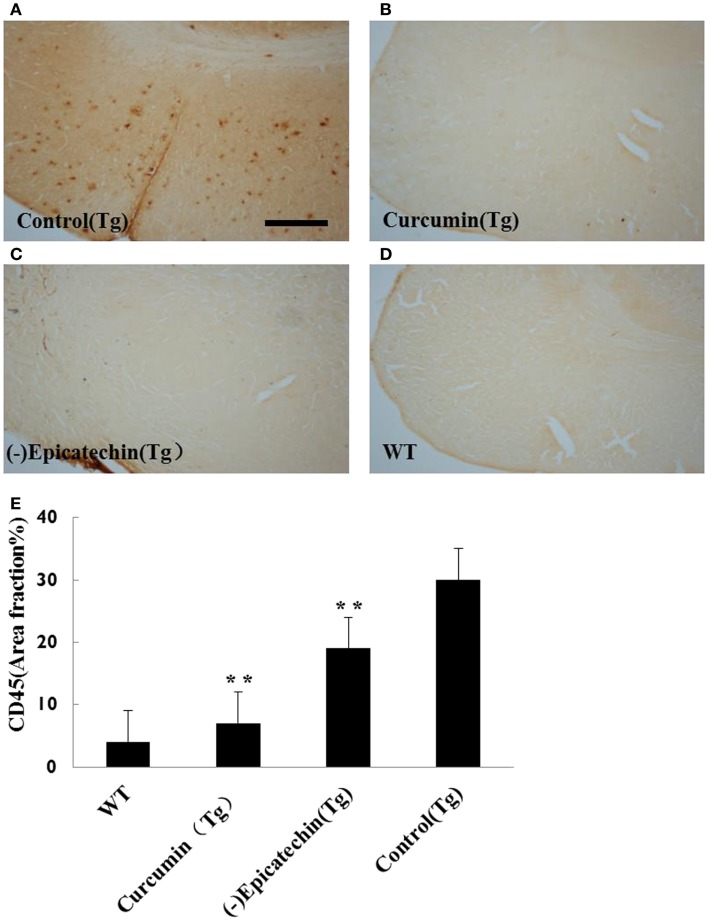
**Effects of (−)epicatechin diet group on microgliosis in the brain**. The area of neocortex and hippocampus was selected for quantification of activated microglia immunostaining. **(A)** Microgliosis in hippocampus and neocortex of APP/PS1 transgenic mice fed with control diet, **(B)** transgenic mouse fed with curcumin, **(C)** transgenic mouse fed with (−)epicatechin, **(D)** wild-type mouse fed with control diet, **(E)** Comparison of CD45 area fraction in neocortex and hippocampus among groups. Statistical analysis showed a significant decrease in the number of microgliosis in the brain treated with (−)epicatechin and curcumin diets. *P* < 0.05 versus APP/PS1 transgenic mice fed with control diet. Scale bar = 0.5 mm. Original magnification, 4×.

**Figure 6:**
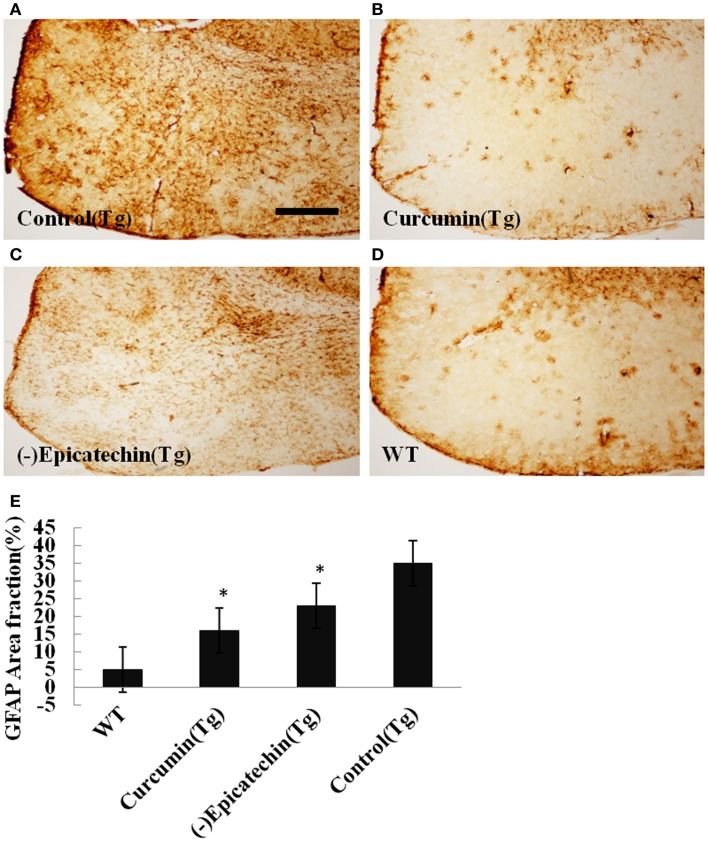
**Effects of (−)epicatechin diet group on astrogliosis in the brain**. The area of neocortex and hippocampus was selected for quantification of astrogliosis immunostaining. **(A)** Astrogliosis in hippocampus and neocortex of APP/PS1 transgenic mice fed with control, **(B)** transgenic mouse fed with curcumin, **(C)** transgenic mouse fed with (−)epicatechin, **(D)** wild-type mouse fed with control diets, **(E)** comparison of GFAP area fraction in neocortex and hippocampus among groups. Statistical analysis showed a significantly decrease in the astrogliosis in the brain treated with (−)epicatechin and curcumin diets. *P* < 0.05 versus APP/PS1 transgenic mice fed with control diet. Scale bar = 0.5 mm. Original magnification, 4×.

The microgliosis (by CD45 antibody) and astrocytosis (using GFAP antibody) in the neocortical and hippocampal regions were investigated by immunohistochemistry. Quantitative analysis followed by one-way ANOVA (*F* = 19.115, *P* < 0.001 and *F* = 6.041, *P* < 0.05) revealed a remarkable differences in levels of microgliosis and astrocytosis between transgenic mice fed with (−)epicatechin diet and animals fed with control diet. The mice consuming (−)epicatechin diet showed significant lower level of microgliosis and astrocytosis by 38 and 35%, but no obvious microgliosis and astrocytosis were observed in the brains of wild-type animals (Figures 5 and 6).

**Figure 7:**
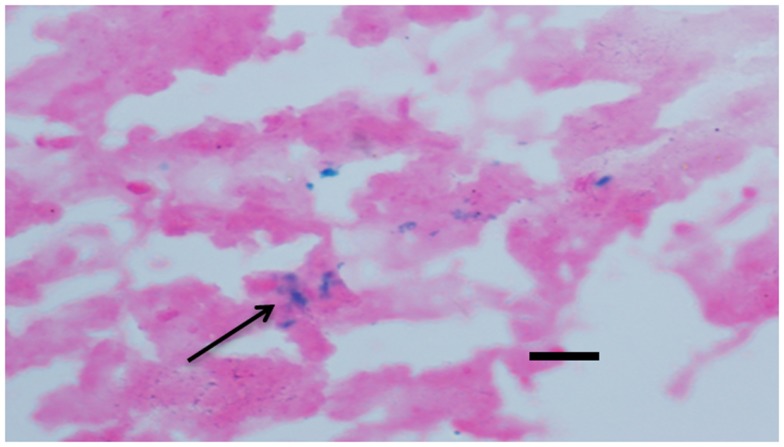
**Effects of (−)epicatechin and curcumin consumption on microhemorrhage profiles**. Microhemorrhage events in the form of the number of Prussian blue-positive profiles were counted, and the average number and standard error of hemosiderin deposits was calculated per each brain hemisphere. Comparison of microhemorrhage profiles per each brain hemisphere among groups. *, **denote *P* > 0.05 versus wild-type littermate fed with Control diet.

Microhemorrhage has been associated with increasing age (16, 17). In AD, MH have been linked with β-amyloid (Aβ) deposition in AD and cerebral amyloid angiopathy (CAA) (18–20). Following the previous method, we detected cerebral MH in brain sections stained with Iron-Prussian Blue staining. After treatment with (−)epicatechin and curcumin diets. MH tended to be lower than transgenic mice fed with control diet, but the difference between groups did not reach statistical significance (Dunnett T3, *P* > 0.05, Figure 7).

### Effects of (−)epicatechin treatment on inflammation cytokines in serum

**Figure 8:**
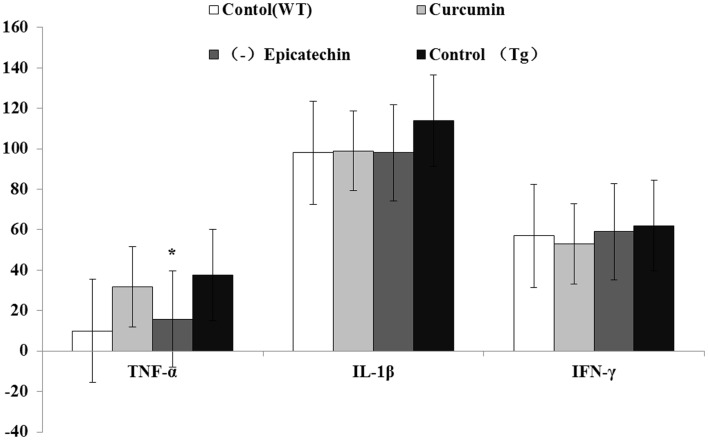
**Effects of curcumin and (−)epicatechin consumption on plasma levels of TNF-a, IL-1β, and IFN-γ**. Levels of TNF-a, IL-1β, and IFN-γ in serum were measured using ELISA kits (eBioscience). Statistical analysis showed the level of TNF-α was significantly decreased in serum of mice (Tg) treated with (−)epicatechin diet. *P* < 0.05 versus APP/PS1 transgenic mice fed with control diet. But levels of IL-1β and IFN-γ did not occur any change in transgenic mice when comparing.

Inflammation is an important hall mark in AD development. Activated microglia mainly release a combination of both pro- and anti-inflammatory cytokines, including IL-1(interleukin-1), IFN-γ (interferon-γ), TNF-α(tumor necrosis factor), and IL-6 (21). Here, we measured levels of the proinflammatory cytokines in the serum using the ELISA Kits. Compared with control (37.6 ± 12.1 pg/ml) and curcumin (30.4 ± 14.2 pg/ml) diet, (−)epicatechin (11.6 ± 2.8 pg/ml) diet significant decreased TNF-α level (*P* < 0.05) in the plasma of transgenic mice (ANOVA TNF-α, *F* = 12.261, *P* < 0.05). No change was found in the cytokines of IL-1and IFN-γ when comparing with control (Tg) diet group (ANOVA IL-1, *F* = 1.428, *P* = 0.274; IFN-γ, *F* = 0.272. *P* = 0.845; Figure 8), and the level of IL-6 in serum was too low to be detected.

## Discussion

The present study suggests long-term oral (−)epicatechin as a food additive can be effective on ameliorating AD pathology in mice. Our results indicate that (−)epicatechin appears to be well tolerated in relation to viability and systemic toxicity by both wild-type and APP/PS1 mice. No adverse event was seen in both strains of mice. (−)Epicatechin significantly inhibited the deposits of amyloid in the brain and reduced the levels of Aβ in the blood and the brain. (−)Epicatechin also attenuated neuroinflammation in APP/PS1 mice, including reducing levels of microgliasis and astrogliosis and lowering the concentration of TNF-α in the plasma of transgenic mice. But (−)epicatechin did not improve learning and memory in APP/PS1 mice after 9 months treatment. This result is different from a previous report showing that the consumption of (−)epicatechin increases memory function of normal mice (22) and this difference can be ascribed to different strains of mice, dosage, and length of treatment.

Soluble Aβ oligomers are major contributors to the toxicity associated with the peptide. The amyloid peptides Aβ40 and 42 are thought to contribute differentially to the disease process (23). It is found that Aβ42 is much more prone to aggregate and more toxic to neurons than Aβ40 (24, 25). We examined Aβ levels in the 2% SDS and formic acid by ELISA. While Aβ42 and 40 levels are decreased in animals fed with (−)epicatechin relative to untreated AD transgenic animals (Dunnett’s T3 Assay, *P* < 0.05), the ratio of insoluble Aβ42/40 was significantly decreased in (−)epicatechin diet fed mice (1.95) relative to fed standard diet mice (2.05).

The decreased ratio (5%) of insoluble Aβ42/40 in (−)epicatechin consumption mice may be attributable to the modulation of β-secretase inhibition. β-secretase is one of important enzymes in the process of APP to Aβ42 (26). However, significant change in the β-secretase activity as measured BACE activity assays, suggests that β-secretase may not play a role in Aβ reduction caused by (−)epicatechin diet. However, we cannot rule out the possibility that the γ-secretase is involved in Aβ metabolism in (−)epicathechin treated mice, γ-secretase is a large multimeric membrane-bound protein composed of presenilins (PS1), nicastrin, and Aph-1. Mutations in three different genes, APP and presenilin-1 and -2 (PS1 and PS2), are known to cause early onset familial AD (27, 28). For this reason, γ-secretase has been considered as a plausible molecular target as a means to interfere with the production of Aβ. Inhibiting γ-site cleavages of γ-secretase is a more attractive approach, which using an APP/Aβ-binding small molecular compound can achieve allosteric modulation of γ-secretase activity and attenuate the Aβ42/40 ratio (29), additional work is required to understand if (−)epicatechin directly target the γ-secretase.

(−)Epicatechin is a flavonoid, possesses free radical scavenging activity and superoxide dismutase activity (30–32). (−)Epicatechin is able to traverse the blood–brain barrier after oral ingestion (33, 34), so it is possible that Aβ neurotoxicity be blocked by an antioxidant mechanism, and by inhibition of Amyloid-beta oligomers and/or fibril formation (35).

Inflammation is considered a major pathological aspect of AD. As a part of the inflammatory response, activated astrocytes and microglia are characteristically found in abundance in the plaques. Besides, AD brains show increased expression of several proinflammatory cytokines such as IL-6, IL-1β, and tumor necrosis factor-α (TNF-α), which are hardly detectable in normal brains (36–39). Increased levels of these cytokines have been described not only in the brains but also in blood and cerebrospinal fluid from AD patients (40).

In this study, our results showed the chronic (−)epicatechin consumption effectively alleviated microgliosis and astrogliosis, and reduced level of TNF-α among transgenic mice. This is consistent with the fact that inhibition of TNF-α by various means reduces AD-like pathology in transgenic AD mice (41, 42). In addition, the levels of IL-1β and IFN-γ in serum did not change and the level of IL-6 was too low to be detected. The data are consistent with our previous studies (13), indicating (−)epicatechin can suppress inflammation in the mouse model of AD.

Although Aβ is the initial factor of the AD pathogenesis, recent efforts to reduce Abeta production or clear Abeta deposition in the brain did not success in clinical trials, such as beta- or gamma-secretase inhibitor and immunotherapies (43). The lessons learnt from these failures suggest that an effective therapy should simultaneously target different aspects of AD pathogenesis, as AD is a multifactorial disease. Neuroinflammation and oxidative stress are key components of AD pathogenesis. Although they are secondary to Abeta deposition, they play critical roles in promoting AD development and progress, via increasing Abeta production, enhancing Abeta deposition, and cause other pathological events such as tau hyperphosphorylation, neuronal degeneration, and death. In this regard, polyphenols possess their own advantages. So far, some polyphenols have been suggested to be able to reduce Abeta production, inhibit Abeta aggregation, attenuate neuroinflammation and oxidative stress, ameliorate tau hyperphosphorylation (44–46). Thus, polyphenols possibly represent a group of natural drug candidate for AD.

In summary, our research confirms the efficacy of (−)epicatechin in APP/PS1 mice AD, but the impact of (−)epicatechin on tau pathology is not clear, also the mechanism needs further research.

## Conflict of Interest Statement

The authors declare that the research was conducted in the absence of any commercial or financial relationships that could be construed as a potential conflict of interest.

## Abbreviations

FA, formic acid; HPLC, high-performance liquid chromatography; IFN-γ, interferon-γ; IL-1, interleukin-1; PCR, polymerase chain reaction; SDS, sodium dodecyl sulfate; TBS, tris buffered saline; TNF-α, tumor necrosis factor alpha.
